# Identification of hypervirulent *Klebsiella pneumoniae* based on biomarkers and Galleria mellonella infection model

**DOI:** 10.1186/s12866-023-03124-0

**Published:** 2023-11-29

**Authors:** Dongmei Mai, Anqi Wu, Ran Li, Donghao Cai, Huichun Tong, Nan Wang, Junqing Tan

**Affiliations:** 1 Guangdong Provincial Second Hospital of Traditional Chinese Medicine, Guangzhou, China; 2 Guangzhou Nanfang College, Guangzhou, China; 3 Guangdong Key Laboratory of Non-Human Primate Research, Guangdong-Hongkong-Macau Institute of CNS RegenerationJinan University, Guangzhou, China

**Keywords:** Hypervirulent *Klebsiella pneumoniae*, Virulence gene, Siderophores, Galleria mellonella infection model

## Abstract

**Background:**

Currently, clinical laboratories lack an effective method to differentiate between classical *Klebsiella pneumoniae* (cKP) and hypervirulent *Klebsiella pneumoniae* (hvKP) strains, leading to delays in diagnosing and treating hvKP infections. Previous studies have identified *peg-344*, *iroB*, *iucA*, _*p*_*rmpA*, _*p*_*rmpA2*, and siderophores (SP) yields greater than 30 μg/ml as reliable markers for distinguishing hvKP from cKp strains. However, these diagnostic tests were conducted on a relatively small study population and lacked sufficient clinical data support. In this study, hvKP strains were identified by biomarker analysis and the Galleria mellonella model. Combined with in vitro and in vivo experiments, the reliability of clinical identification method of hvKP was verified, which provided an experimental basis for timely diagnosis of hvKP infection.

**Results:**

According to the clinical data, a total of 108 strains of hvKP were preliminary screened. Among them, 94 strains were further identified using PCR analysis of biomarkers and quantitative determination of SP. The high virulence of hvKP was subsequently confirmed through infection experiments on Galleria mellonella. Additionally, susceptibility testing revealed the identification of 58 carbapenem-resistant hvKP (CR-hvKP) strains and 36 carbapenem-sensitive hvKP (CS-hvKP) strains. By comparing molecular diagnostic indexes, molecular characteristics such as high SP production of CR-hvKP were found.

**Conclusion:**

The combination of clinical data and molecular diagnostic index analysis effectively enables the identification of hvKP, particularly CR-hvKP. This study provides a scientific basis for accurate clinical identification and timely treatment of hvKP.

**Supplementary Information:**

The online version contains supplementary material available at 10.1186/s12866-023-03124-0.

## Background

*Klebsiella pneumoniae* (KP) is a common conditional pathogen in clinical practice, and its emergence of drug resistance has become a major problem plaguing the clinic. At present, two pathogenic types of classical *Klebsiella pneumoniae* (cKP) and hypervirulent *Klebsiella pneumoniae* (hvKP) have been clinically identified [1, 2]. hvKP is a highly virulent variant of cKP that can infect from a distance, and can be life-threatening in severe cases. Due to the strong pathogenicity, disability and mortality rate of hvKP, its serious harm has attracted great clinical attention. Most cases of hvKP infection are known to be community-acquired, and there are increasing reports of hvKP infection occurring in healthcare settings [3] and resistance is increasingly common [4, 5]. With the widespread use of carbapenem antibiotics in clinical practice, more and more cases have been found in recent years about carbapenem-resistant hypervirulent *Klebsiella pneumoniae* (CR-hvKP) in China [6]. No significant difference was found between CR-hvKP and cKP in the growth state, suggesting that the adaptation cost of CR-hvKP is limited, but the high toxicity and drug resistance of CR-hvKP increase clinical mortality, which poses significant challenges to public health and infection control [7, 8].

At present, there is no unified standard for the identification of hvKP at home and abroad. Suspicion by experienced clinicians is the primary route of consideration for hvKP and is not suitable for strains that lack clinical data. Furthermore, the string test is frequently employed in clinical settings as an initial means of differentiating hvKP strains. However, it should be noted that the string test's specificity is limited, as not all hvKP strains exhibit a pronounced mucoid phenotype. This characteristic can also be observed in cKP strains [9, 10]. Current hvKP detection methods include polymerase chain reaction (PCR) and multilocus sequence typing (MLST), pulse field gel electrophoresis (PFGE) and proteomic methods, which are complex, time-consuming, and require technical competence.

Preliminary sequencing of hvKP strains in previous studies revealed the presence of large and highly similar virulence plasmids pK2044 (224,152 bp) and pLVPK (219,385 bp) [11, 12], which encode genes that confer a hypervirulence phenotype. Such as *iuc* (siderophore aerobic actin biosynthesis gene) [13], *iro* (salmonella biosynthesis gene) [14], *peg-344* (a metabolic transporter of unknown function) [15], *rmpA* and *rmpA2* (a regulator that promotes capsule production) [16]. In addition, hvKP possesses a high iron trapping capacity and is mediated by *rmpA*/*rmpA2*-mediated capsular polysaccharide overproduction, which confers high viscosity and is associated with mucus viscosity-associated gene A (*magA*). Therefore, studies have found *peg-344*, *iroB*, *iucA*, _*p*_*rmpA*, _*p*_*rmpA2* and siderophores (SP) yield greater than 30 μg/ml, which have been shown to accurately distinguish hvKP from cKp strains [10], but the study population is relatively small and not supported by more clinical data. In addition, in recent years, the Galleria mellonella infection model as a pathogenic bacteria virulence detection test has attracted everyone's attention [17–19], because the natural immune system of the Galleria mellonella is similar to that of mammals, with enzymes, reactive oxygen species and bacitracin required to protect bacterial infection, its blood cells show phagocytosis and bactericidal functions similar to mammalian neutrophils and macrophages, and can survive in a 37 °C environment, so the Galleria mellonella model is considered to be more suitable for the study of human pathogenic bacteria, which can provide in vivo test data for pathogenic drug treatment. The Galleria mellonella model infected with hvKP was established to investigate the infection characteristics of this pathogen in the host. This research provided valuable insights into the host–pathogen interaction, which can contribute to improved clinical diagnosis and treatment strategies.

This study aimed to enhance the clinical identification technology for hvKP infections by integrating clinical data analysis with in vitro and in vivo experiments. The diagnostic techniques employed included PCR analysis of virulence genes, SP detection, and the use of the Galleria mellonella infection model to confirm the heightened virulence characteristics of hvKP strains. By utilizing these comprehensive diagnostic tools, the study sought to provide clinicians with effective means to accurately identify hvKP infections and guide appropriate treatment strategies for patients infected with hvKP.

## Result

### Initial screening of hvKP clinical isolates

Based on previous studies [20], we set clinical inclusion criteria for initial screening of hvKP, and used clinical data to preliminarily screen 94 hvKP strains (Table 1). Possible primary infections (number of cases) are liver abscess (4), renal abscess (4), lung abscess (3), abdominal wall abscess (1), testicular abscess (1), perianal abscess (1), acute cholecystitis (23), and necrotizing fasciitis (2).

**Table 1:** Clinical data of hvKP

Serial number	Strain number	Specimens	Site of primary infection	Transfer or other parts
1	2	purulence	liver	gall
2	5	sputum	gall	lung
3	7	Venous catheter blood	lung	
4	10	urine	gall	bladder
5	12	sputum	gall	lung
6	14	Perinephric space effusion	kidney	
7	15	sputum	lung	
8	16	sputum	peritoneum	lung
9	17	sputum	lung	
10	19	sputum	gall	lung
11	20	sputum	lung	
12	21	sputum	gall	lung
13	22	sputum	gall	lung
14	23	sputum	gall	lung
15	25	venous blood	inguen	
16	26	sputum	gall	kidney
17	28	sputum	lung	
18	29	sputum	eye	lung
19	30	sputum	pancreas	lung
20	31	secreta	abdomen	lung
21	32	urine	soft tissue	
22	33	sputum	brain	lung
23	34	urine	soft tissue	
24	35	Wound secretion	testis	
25	36	sputum	lung	
26	37	sputum	gall	lung
27	38	Central venous blood	liver	gall
28	39	sputum	lung	
29	40	sputum	lung	
30	41	Central venous blood	lung	
31	42	urine	gall	bladder
32	43	sputum	lung	
33	44	Alveolar lavage fluid	lung	
34	46	sputum	lung	
35	47	urine	pancreas	kidney
36	48	urine	testis	
37	49	pus	gall	
38	50	Central venous blood	lung	
39	51	urine	kidney	
40	52	sputum	liver	lung
41	53	sputum	lung	
42	54	sputum	lung	
43	55	sputum	lung	
44	56	venous blood	gall	
45	57	urine	gall	kidney
46	58	sputum	lung	
47	59	venous blood	kidney	
48	60	sputum	lung	
49	61	urine	peritoneum	kidney
50	62	sputum	lung	
51	63	sputum	lung	
52	64	pus	napes	
53	65	sputum	soft tissue	lung
54	67	Venous catheter blood	lung	
55	68	sputum	lung	
56	70	secreta	heart	
57	71	Central venous blood	lung	
58	72	venous blood	lung	
59	73	urine	gall	kidney、bladder
60	74	sputum	lung	
61	75	drain	liver	
62	76	urine	lung	
63	77	sputum	kidney	lung
64	78	sputum	lung	
65	79	urine	intestines	
66	80	venous blood	kidney	
67	81	urine	lung	
68	82	sputum	lung	
69	83	sputum	lung	
70	84	venous blood	lung	
71	85	venous blood	lung	
72	86	urine	bladder	
73	87	sputum	lung	
74	88	Venous catheter blood	stomach	
75	89	urine	urethra	
76	90	pus	perianal region	
77	91	sputum	lung	
78	92	secreta	ear	lung
79	93	sputum	lung	
80	94	sputum	lung	
81	95	sputum	lung	
82	96	sputum	kidney	lung
83	97	sputum	lung	
84	98	sputum	lung	
85	99	venous blood	kidney	
86	100	urine	gall	
87	101	sputum	gall	
88	102	venous blood	epididymis	
89	103	urine	lung	
90	104	secreta	kidney	
91	105	sputum	gall	
92	106	venous blood	lung	
93	107	venous blood	lung	
94	109	sputum	gall	lung、pancreas

### Identification of hvKP clinical isolates

It has been found that *peg-344*, *iroB*, *iucA*, _*p*_*rmpA*, _*p*_*rmpA2* and SP yields greater than 30 μg/ml have been shown to accurately distinguish hvKP from cKp strains [10]. Therefore, this study established hvKP identification criteria: initial screening of hvKP strains that are positive for any gene such as *peg-344*, *iroB*, *iucA*, _*p*_*rmpA*, _*p*_*rmpA2* or SP yield greater than 30 μg/ml, were identified as hvKP, and vice versa, cKP.

#### String test of initial screening hvKP strains

**Fig. 1:**
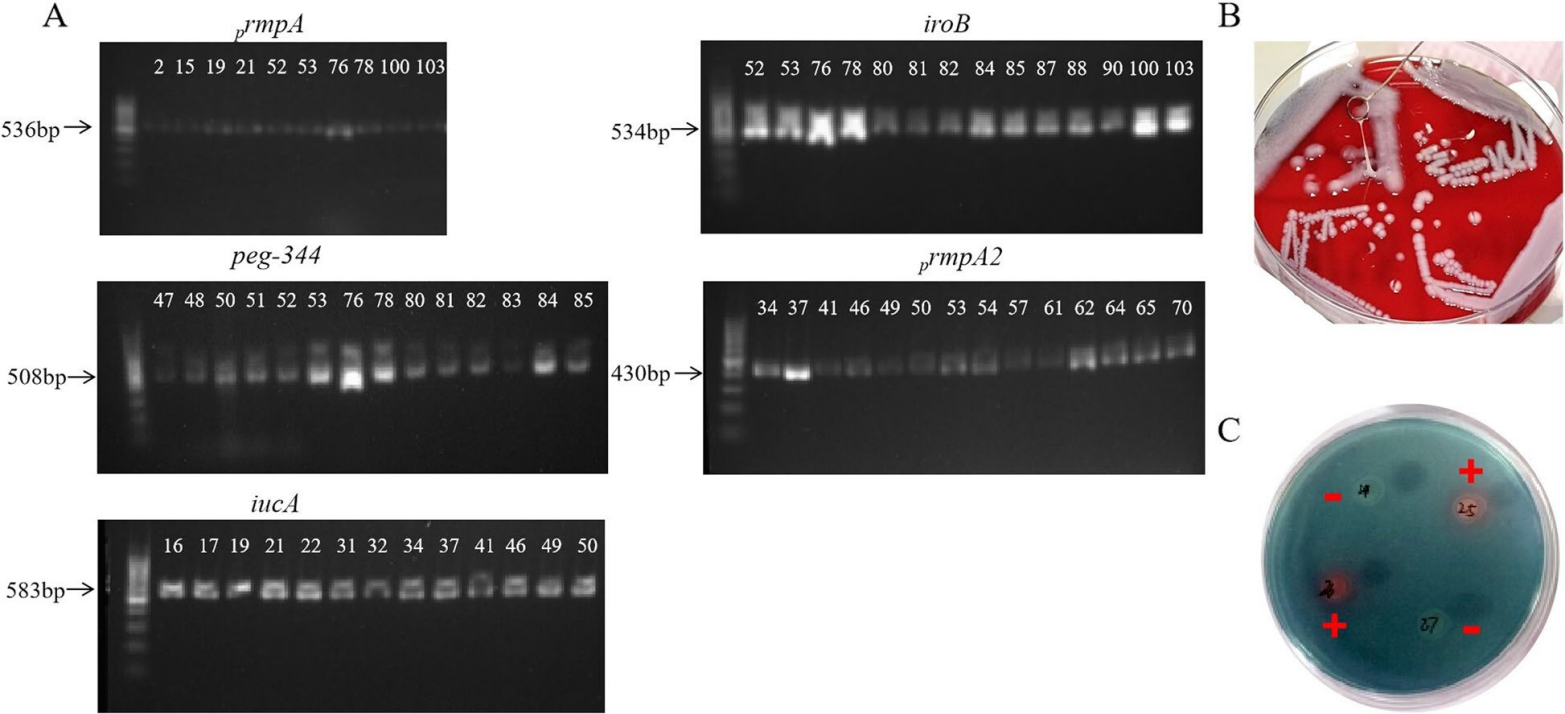
Results of each diagnostic index of hvKP (**A**), Southern blot analysis of virulence genes of some primary screened hvKP isolates (**B**), string test of initial screening hvKP strains (**C**), SP qualitative testing

In this study, 13 of the 108 primary hvKP strains were positive for string test, and the positive string results were shown in Fig. 1B.

#### Virulence gene detection of initial screening hvKP strains

In this study, PCR was used to detect five virulence genes (*peg-344*, *iroB*, *iucA*, _*p*_*rmpA*, _*p*_*rmpA2*) in 108 primary screening hvKP strains. Among them, 74 strains were positive for any of these five virulence genes, and the part of positive results were shown in Fig. 1A, of which 41 cases were detected with *peg-344*-positive strains, 18 cases with *iroB*-positive strains, 47 cases with *iucA*-positive strains, 10 cases with _*p*_*rmpA*-positive strains and 47 cases with _*p*_*rmpA*2-positive strains. The details of southern blots were shown in Figure S1.

#### SP detection of initial screening hvKP strains

##### SP qualitative testing

The production of SP of primary screening hvKP strains was detected by CAS medium. 59 SP-positive strains were detected in 108 hvKP strains, with an orange halo around the colonies (Fig. 1C). However, the diameter and size of the rings vary, indicating that the strain can produce SP, but the amount of production varies.

##### SP quantitative detection

**Fig. 2:**
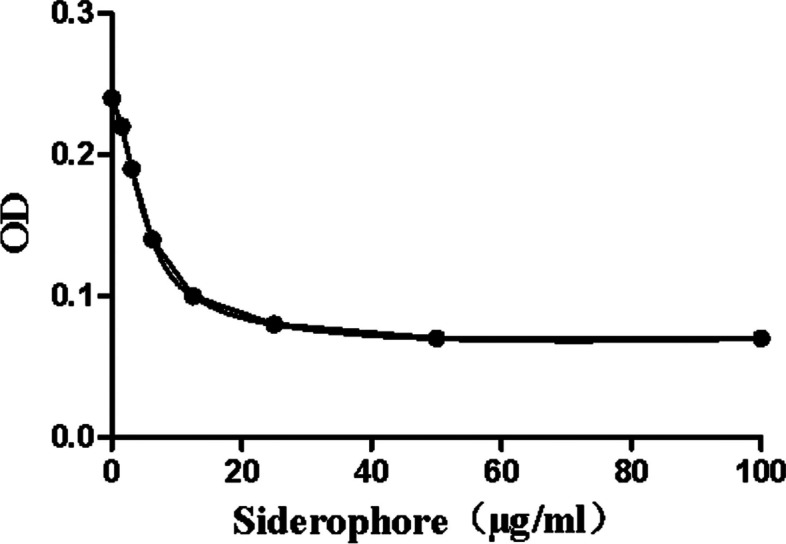
SP quantitative standard curve

The production amount of SP was determined in 59 strains that tested positive in the qualitative SP testing using a quantitative SP test. Deferoxamine mesylate was employed as the standard, and a quantitative standard curve for SP was constructed, as depicted in Fig. 2. The calculated SP yield exceeded 30 μg/ml (Table S1).

Therefore, according to the hvKP identification criteria set by this study, a total of 94 hvKP strains were screened, and the detection rate of each diagnostic index of hvKP was analyzed, as shown in Table 2, the positive rates of _*p*_*rmpA, iroB, peg-344, *_*p*_*rmpA2, iucA* were 10.64%, 19.15%, 43.62%, 50%, and 50%, respectively. The positive rate of string test was 13.83%, and the positive rate of SP test was 62.77%. It can be seen that the positive rate of string test is low, and the missed rate of hvKP is high, which cannot be used for the identification of hvKP. The detection rate of *peg-344*, _*p*_*rmpA2*, *iucA* and SP tests in virulence genes was high, all > 40%, which was a key indicator for the diagnosis of hvKP.

**Table 2:** Positive rate of hvKP diagnostic index (%)

Diadynamic criteria	Positive rate (%)
_*p*_*rmpA*	10.64
*iroB*	19.15
*peg-344*	43.62
_*p*_*rmpA2*	50.00
*iucA*	50.00
String test	13.83
SP test	62.77

#### Experiment on virulence detection of Galleria mellonella

##### Galleria mellonella health index score

In this study, a combination of PCR analysis of virulence genes and SP detection was employed.. If either of the two tests yielded a positive result, the high virulence characteristics of hvKP were verified by the virulence test of Galleria mellonella. Therefore, 94 hvKP strains were screened out from the above experiments for the virulence detection of Galleria mellonella, 94 hvKP strains were prepared into 1 × 10^5^、1 × 10^6^、1 × 10^7^、1 × 10^8^ CFU/ml bacterial solutions by double dilution method, respectively. The Galleria mellonella were inoculated with phosphate buffer saline (PBS) buffer and blank group as the parallel controls. There were 6 groups with 10 larvae in each group. The health of larvae was assessed at 6 h, 12 h, 24 h, 48 h, 72 h, 96 h, 120 h and 144 h after inoculation. The assessment involved recording the activity level, cocoon formation, blackening, and overall survival of the Galleria mellonella larvae. The scoring criteria for the specific larval health index were adopted from a previous study [21].

**Fig. 3:**
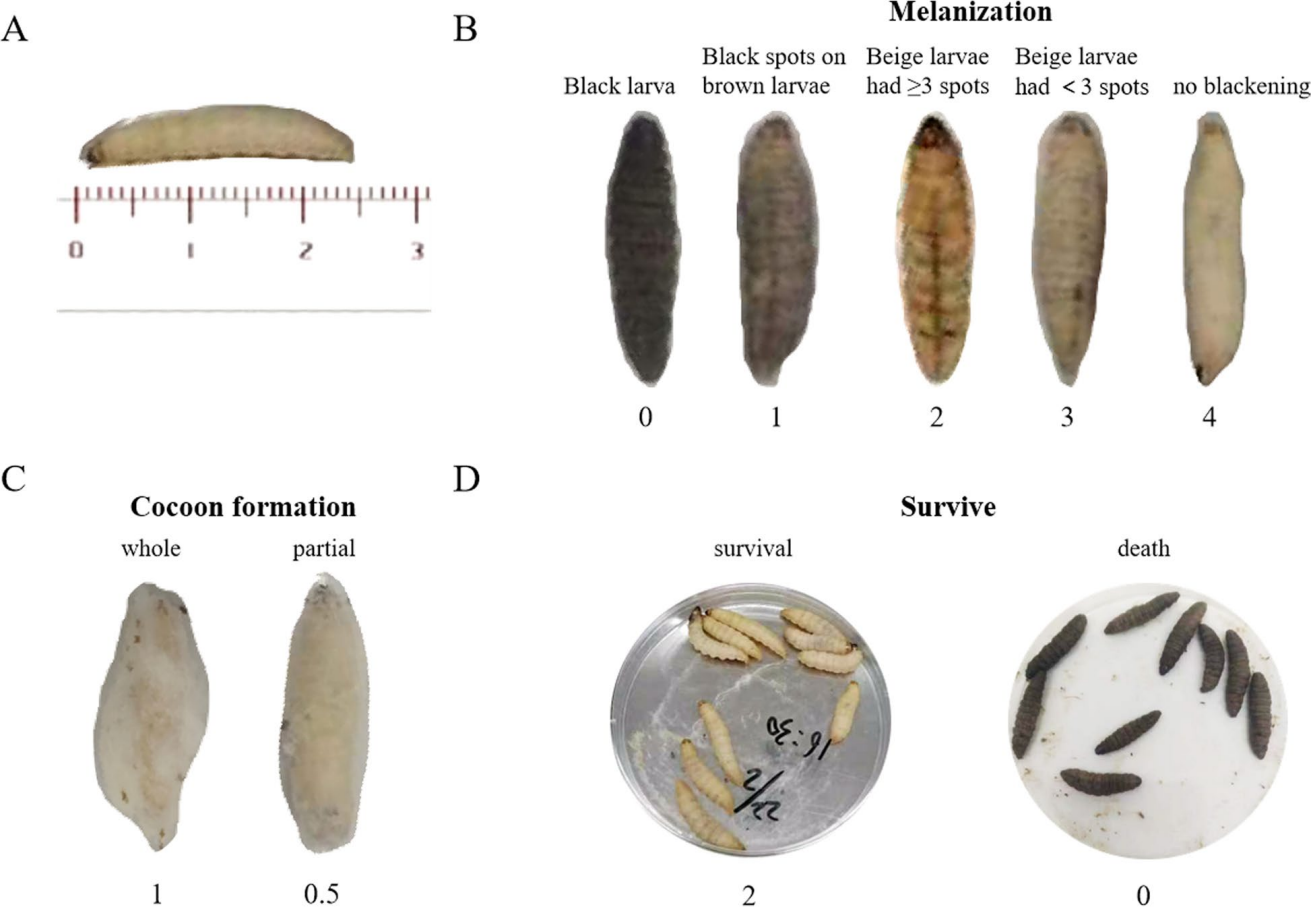
Performance of Galleria mellonella at different ratings (**A**), The mean length of Galleria mellonella was 2.5cm (**B**), melanization of Galleria mellonella (**C**), cocoon formation of Galleria mellonella (**D**), survival of Galleria mellonella

As shown in Fig. 3A, the Galleria mellonella used in this study were 2 ~ 3 cm long, and were divided into 5 situations according to the formation of larval melanin (Fig. 3B), with a score of 0 ~ 4. According to the larval cocoon formation, it was divided into no cocoon, partial cocoon and whole cocoon, with a score of 0 ~ 1 (Fig. 3C). According to the survival of the larvae, the score was 0 and 2, in which the death of the larvae was black and no response to touch (Fig. 3D).

**Fig. 4:**
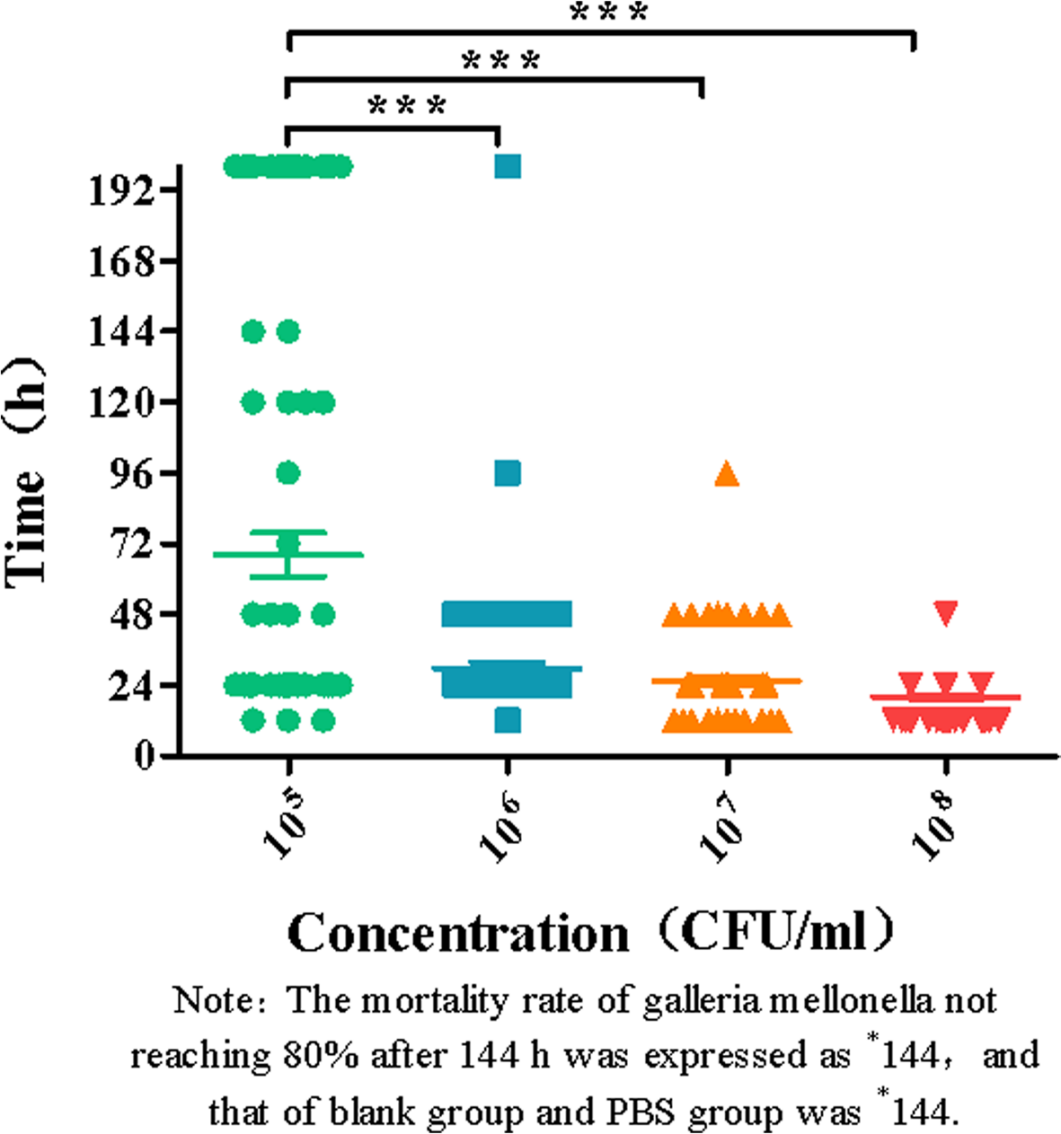
Health index scores of hvKP strains in Galleria mellonella at different concentrations and different time

The Galleria mellonella were divided into six groups. The experimental group was the larvae injected with four different concentrations of hvKP bacterial solution (1 × 10^5^–1 × 10^8^ CFU/ml), the control group was the blank group without treatment and the PBS group was injected with PBS. The average of 10 larvae in each group was taken and compared according to the x ± s score of each group. As shown in Fig. 4, it was found that after injection of hvKP strains with different concentrations, the larvae of the Galleria mellonella showed a sharp decrease in health index score within 6 to 24 h, and the number of surviving larvae decreased significantly after 24 h. The larval scores of the high-concentration group were significantly lower compared to those of the low-concentration group. Additionally, as the concentration of the bacterial solution increased, the larval scores decreased within the same time period. This concentration-dependent trend in the larval scores indicated that the 94 strains of hvKP exhibited high toxicity characteristics. On the other hand, the scores of the blank group and the PBS group did not show significant changes over the observation period, suggesting that these groups did not experience any adverse effects.

##### Time of death of Galleria mellonella

**Fig. 5:**
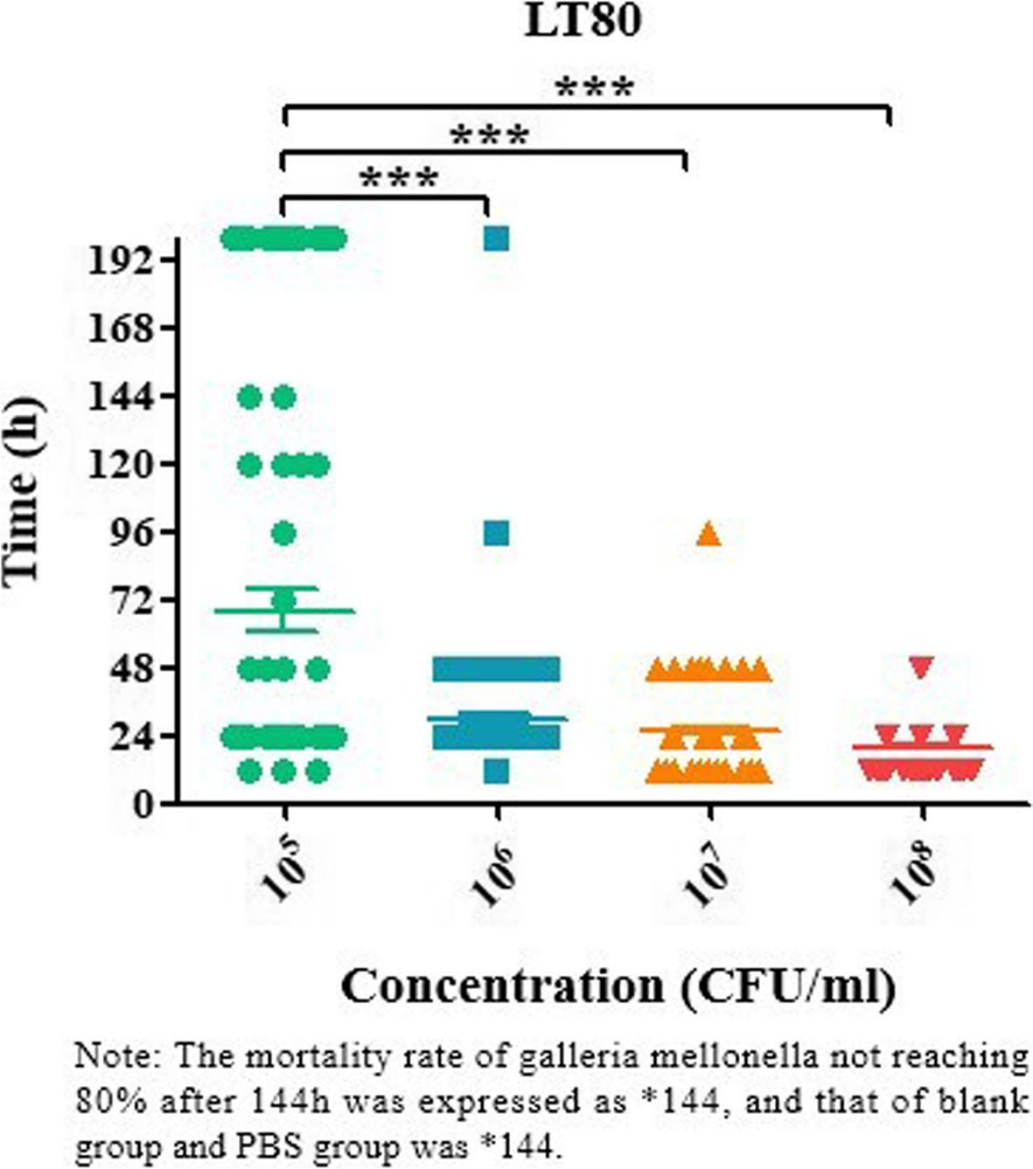
Time to 80% Galleria mellonella death of hvKP strains at different concentrations (LT80)

After inoculation of 94 Galleria mellonella larvae with different concentrations (1 × 10^5^, 1 × 10^6^, 1 × 10^7^, 1 × 10^8^ CFU/ml), the time when the larvae reached 80% death (LT80) during 6 to 144 h was recorded (Fig. 5). Among them, the survival time of larvae inoculated by the low concentration group (1 × 10^5^ and 1 × 10^6^ groups) was > 144 h, which was labeled as *144 h, while most of the larvae inoculated in the high concentration group (1 × 10^7^, 1 × 10^8^ groups) reached 80% death within 48 h. With the increase of the concentration of bacterial solution, the death time of 80% larvae was shorter and showed a concentration dependence. Compared with the low concentration group (1 × 10^5^ group), the 1 × 10^6^, 1 × 10^7^ and 1 × 10^8^ groups had significant statistical differences (*P* < 0.0001). However, 80% of the larvae in the blank group and PBS group did not die with the increase in observation time, so LT80 at different times was expressed as *144 h. Therefore, it was further verified that all 94 hvKP strains had high toxicity characteristics.

##### Survival curve of Galleria mellonella

**Fig. 6:**
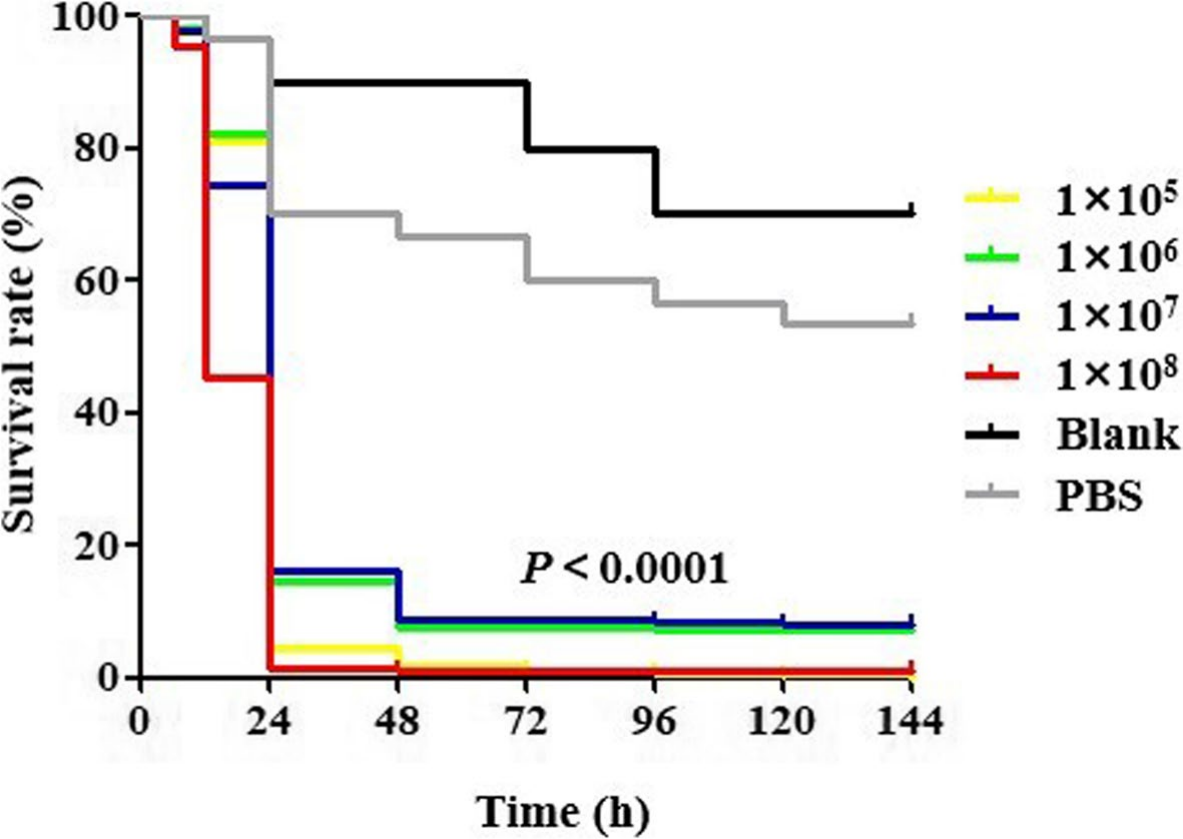
Survival curves of Galleria mellonella in different concentrations of hvKP strains

Figure 6 illustrates the survival curves of Galleria mellonella larvae that were injected with different concentrations of hvKP strains, as well as the PBS and blank control groups. The survival rate of the larvae inoculated with different concentrations of hvKP strains was significantly lower compared to the control group. This difference in survival rates was found to be statistically significant(*P* < 0.0001). It was further verified that all 94 hvKP strains had high virulence characteristics.

### Molecular characteristics of the CR-hvKP strain

Worldwide, there are increasing reports of antibiotic-resistant hvKP isolates, mainly in countries with endemic hvKP transmission [22]. Studies have found that the hvKp strain obtains a carbapenem-resistant plasmid and evolves into CR-hvKP [23], and the characteristics of high virulence and multidrug resistance have attracted more and more clinical attention. Therefore, while observing the clinical data of patients, according to the drug susceptibility report of the isolates, 36 strains of carbapenem-sensitive hypervirulent *Klebsiella pneumoniae* (CS-hvKP) and 58 strains of CR-hvKP were found in 94 hvKP strains, and their molecular characteristics were comparatively analyzed. The comparison of various diagnostic indicators is shown in Table 3. We found that compared with CS-hvKP, the detection rate of *peg-344*, _*p*_*rmpA* and *iroB* virulence genes in CR-hvKP was lower with a statistically significant difference (*P* < 0.00), and the detection rate of _*p*_*rmpA2*, iucA was higher with a statistical difference (*P* < 0.05), while the string test and SP experiment did not have a statistical difference (*P* > 0.05). The virulence genes _*p*_*rmpA2* and *iucA* may be involved in plasmid integration with carbapenems resistance genes, carried by hvKp or CRKP clonal strains, and thus evolved into CR-hvKP.

**Table 3:** Comparison of the positive rate of each diagnostic index in CS-hvKP group and CR-hvKP group (%)

Diadynamic criteria	Group		*χ*^2^	*P*
	CS-hvKP (*n* = 36)	CR-hvKP (*n* = 58)		
_*p*_*rmpA*	25.00	1.72	12.66	0.00^***^
*iroB*	41.67	5.17	19.11	0.00^***^
*peg-344*	58.33	34.48	5.14	0.02^*^
_*p*_*rmpA2*	19.44	68.97	21.79	0.00^***^
*iucA*	25.00	65.52	14.59	0.00^***^
String test	22.22	8.62	3.45	0.06
SP test	58.33	65.52	0.49	0.48

Note: * represents *P *< 0.05, *** represents *P *< 0.001

**Fig. 7:**
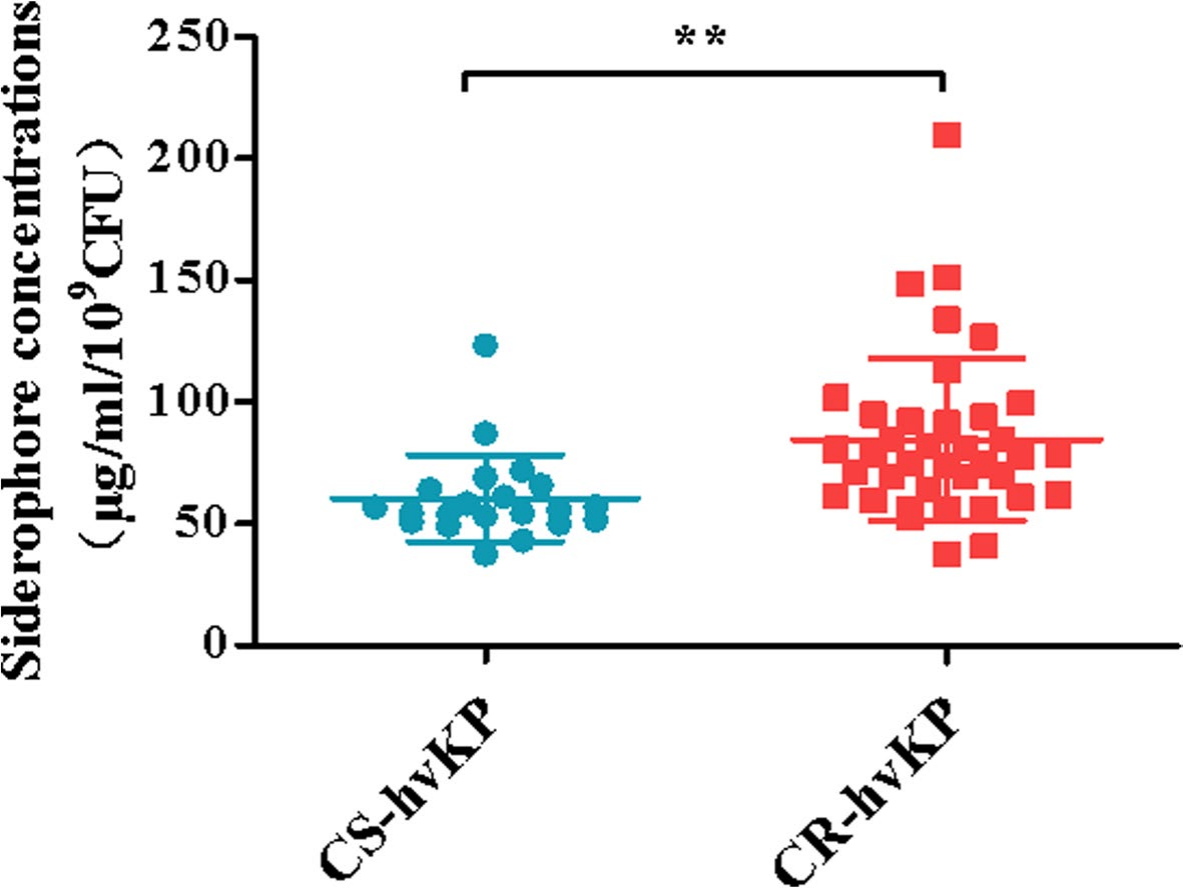
Comparison of SP concentrations of CS-hvKP and CR-hvKP strains

The SP of 59 SP-positive hvKP strains were quantitatively detected. According to the drug resistance of carbapenems, the strains were divided into CS-hvKP and CR-hvKP, and the concentration characteristics of SP were analyzed. As shown in Fig. 7, the CR-hvKP strain was found to have a higher concentration of SP compared to CS-hvKP, and the difference was statistically significant (*P* < 0.01). The results show that CR-hvKP has a stronger iron-bearing capacity.

## Discussion

In recent years, there has been a noticeable increase in the isolation rate of hvKP. This rise in hvKP infections has garnered significant attention due to the associated infectious diseases. hvKP has the potential to cause severe infections, including central nervous system infections. Furthermore, infections caused by hvKP can enhance the development of endophthalmitis in infected patients. These infections pose a serious threat to public health as they are associated with high morbidity and mortality rates. The growing recognition of hvKP's virulence and its impact on human health underscores the need for effective diagnosis, treatment, and prevention strategies. In addition, studies have reported that hvKP strains are resistant to multiple antibiotics [24–26], and the combination of high virulence and high resistance characteristics brings major challenges to clinical treatment. However, the current lack of standardized evaluation criteria for identifying hvKP strains poses a challenge in clinical practice. However, it is crucial to enhance the timely detection of hvKP infections to aid clinicians in promptly considering potential sites of occult infection. Identifying these infectious lesions can guide clinicians towards appropriate interventions such as drainage, targeted treatment, or extended treatment duration. By implementing robust surveillance and diagnostic protocols, healthcare providers can improve their ability to detect and manage hvKP infections effectively. Additionally, ongoing research efforts are necessary to develop consensus guidelines and standardized approaches for the identification and management of hvKP infections.

According to studies, infection with hvKP includes tissue-invasive infections, including clinical syndromes such as liver abscess, endophthalmitis, osteomyelitis, and central nervous system disease, and multiple sites of infection or metastases [27]. Therefore, the clinical inclusion criteria for hvKP in this study were set for preliminary screening of clinical isolates. In addition, 94 hvKP strains were identified among 108 hvKP strains initially screened by virulence gene test, SP experiment and Galleria mellonella infection model. The detection rate reached 87.04% (Table 1), which reflects the feasibility of clinical inclusion criteria. Potential hvKP strains were screened out from a large number of clinical data and further identification was carried out, which greatly shortened the time and cost of identification and was conducive to the timely detection of clinical infection of hvKP.

Most clinics today define hvKP only by detecting high mucus phenotype positivity, but the literature shows that some cKP strains also have high mucus characteristics, and existing reports cannot prove whether all hvKP strains have high viscosity characteristics, indicating that high mucus phenotypes lack specificity. In this study, the positive rate of string test is only 13.83% (Table 2), which is lower than the positive detection rate of 5 virulence genes, so hvKP may be missed according to the mucus phenotype, and hvKP is not rigorous enough to identify hvKP by string test alone. Virulence gene detection and the iron uptake system of KP can be used as important biological detection markers of hvKP [10], and animal experiments can intuitively reflect the virulence of KP in vivo. To mutually verify the identification method of hvKP strains, we conducted in vitro experimental identification by combining virulence gene PCR analysis and the SP test. Additionally, we employed an in vivo experiment using Galleria mellonella as a model to assess virulence. This comprehensive approach allowed us to validate the identification method and enhance our understanding of hvKP's virulence characteristics.

In this study, PCR analysis of five virulence genes was performed on 108 initially screened hvKP clinical isolates, and the positive rates of hvKP virulence genes were detected as _*p*_*rmpA2*, *iucA*, *peg-344*, *iroB* and _*p*_*rmpA* in descending order, and the detection rate of the first three virulence genes was > 40% (Table 2). Studies have revealed that the prmpA2 gene is situated on the plasmid carrier gene within the virulence plasmid p-LVPK. This gene plays a crucial role in promoting the expression of the Klebsiella pneumoniae (KP) capsule gene, thereby facilitating capsule synthesis and the manifestation of a high mucosity phenotype. Deletion of the prmpA2 gene has been shown to decrease capsule production and subsequently reduce both the yield and virulence of the capsule. These findings highlight the importance of the prmpA2 gene in the pathogenicity of hypervirulent Klebsiella pneumoniae strains [16]. iucA is located on the KP virulence plasmid and plays a critical role in the high virulence phenotype as a genetic marker for the identification of hvKP and cKP [14]. *peg-344* is a newly mined virulence gene in recent years, present on hvKP virulence plasmids [15], and studies have shown that *peg-344* is unique to hvKP and can be used for rapid diagnostic tests for hvKP [10]. The use of these biomarkers has high accuracy for the clinical diagnosis of hvKP and is defined as the most accurate molecular marker for hvKP. Therefore, the top three virulence genes _*p*_*rmpA2, iucA*, *peg-344* with high detection rates found in this study can be used as key indicators for the diagnosis of hvKP.

At present, the virulence factors affecting the pathogenicity of hvKP include SP in addition to capsular polysaccharides [14]. Data suggest that hvKP strains have the ability to produce higher bioactive iron-trapping molecules than cKP strains [2], which contributes to virulence and pathogenesis, helps host defenses or increases the efficiency of bacterial iron acquisition. hvKP secretes four hemophores [28], including aerobacillin, salmonectin, enteromycetin, and yersin. Among them, aerobacillin is the most important virulence factor and can carry iron from other host tissue cells. Therefore, the production of aerobacillin accounts for more than 90% of the total hephrophobin activity, and hvKP is an invasive strain that is more likely to secrete aerobacillin, so aerobacillin is mainly related to the serious infection caused by hvKP [29]. In this study, the positive rate of SP qualitative test reached 62.77% (Table 2), which shows that SP experiment plays an important role in identifying hvKP. In addition, quantitative SP concentrations of > 30 μg/mL have been found to have a predictive effect on hvKP strains compared to cKP strains [20]. Therefore, in this study, a quantitative test was added to the positive SP qualitative experiment, and the SP concentration was > 30 μg/ml (Table S1), which further confirmed the feasibility of SP experiment for the identification of hvKP. In summary, SP > 30 μg/ml is of great significance as a diagnostic index for hvKP.

In addition, studies have reported that combined with the virulence level test of Galleria mellonella, it can improve the sensitivity, specificity, and positive predictive value of hvKP [30]. In this study, 94 hvKP strains positive in SP test or PCR for virulence gene were detected by establishing a model of infection of Galleria mellonella. The experimental results demonstrated a significant difference in the health index scores, time of death, and survival curves of larvae injected with varying concentrations of hypervirulent Klebsiella pneumoniae (hvKP) bacteria compared to those injected with PBS and the blank control (As shown in Figs. 4, 5, and 6). These findings further confirmed the high virulence characteristics of hvKP.. Therefore, from the results of in vitro and in vivo experiments, it can be concluded that the identification criteria for hvKP in this study are feasible.

In general, hvKP has high virulence and low drug resistance, but hvKP shows increased drug resistance under the action of long-term selective pressure of clinical drugs, resulting in more and more cases of CR-hvKP in recent years. The superbugs formed by the combination of virulence and drug resistance need to be paid great attention by clinicians, and it is of great significance to understand the clinical characteristics of CR-hvKP, formulate infection control measures, and effectively prevent its spread. In this study, 36 strains of CS-hvKP and 58 strains of CR-hvKP were found through susceptibility experiments. In order to further study the molecular characteristics of CR-hvKP, this study compared the molecular characteristics of CR-hvKP, and found that the detection rate of the diagnostic indicators _*p*_*rmpA2, iucA* and SP tests of CR-hvKP was higher, and the detection rate of *peg-344*, _*p*_*rmpA* and *iroB* virulence genes was lower and there were statistical differences (*P* < 0.05) (Table 3). The virulence factor of CR-hvKP is known to play an important role in its pathogenesis, and hvKP can evolve into CR-hvKP if exogenous carbapenemase-coding plasmids are accidentally obtained during host-to-host evolution and transmission [7, 31, 32]. Therefore, we suspect that the _*p*_*rmpA2* and *iucA* virulence genes may be involved in plasmid integration with carbapenem resistance genes, carried by hvKP or CRKP clonal strains, and thus evolved into CR-hvKP. Previous studies have shown that hvKP strains have the ability to produce larger, more vigorous iron-trapping molecules than cKP strains and are primarily associated with severe infections caused by hvKP [29]. The study results revealed a statistically significant difference in the concentration of SP between CR-hvKP strains and CS-hvKP strains (as shown in Fig. 7). This finding suggests that CR-hvKP exhibits a higher capacity to form SP, indicating a potentially heightened threat of clinical infection compared to CS-hvKP. As a region with a high infection rate of hvKP, China needs to implement strict infection control measures, and an in-depth analysis of the mechanism of hvKP resistance to carbapenems is of great significance to reduce the emergence and spread of CR-hvKP.

## Conclusion

The clinical inclusion criteria established in this study for hvKP demonstrate a high detection rate, enabling preliminary screening of hvKP based on patient symptoms. Virulence gene detection of 108 hvKP strains revealed that *peg-344*, *prmpA2*, and *iucA* genes exhibited high detection rates, indicating their significance as key identification genes for hvKP strains. Additionally, hvKP demonstrated a robust ability to acquire iron ions, with an SP level greater than 30 μg/ml serving as a predictive indicator, thus becoming an important diagnostic criterion for hvKP. Furthermore, the establishment of a Galleria mellonella virulence detection model successfully validated the heightened virulence of hvKP, providing a foundation for further in vivo research on hvKP. Moreover, the identification of CR-hvKP strains with both high virulence and drug resistance characteristics highlights the importance of studying the acquisition mechanisms of their virulence and drug resistance genes. Such investigations hold promise for inhibiting drug resistance spread and facilitating the development of new treatments.

## Materials and methods

### KP identification

MALDI-TOF MS method was used to identify KP, which was carried out on the EXS3000 automatic microbial mass spectrometry detection system (Zhongyuan Huiji Company, Chongqing, China). Sample collection is done by collecting a portion of the colony from columbia blood plate medium (Dijing Microbial Technology Company, Guangzhou, China) and placing it on a MALDI-TOF target plate. Each deposit on the target plate was covered with 1μL of matrix solution (α-cyano-4-hydroxycinnamic acid) and air-dried. After sample preparation, for each target slide, instrument calibration was performed using the *E. coli* reference strain ATCC 8739 according to the manufacturer's instructions. The spectrum is obtained according to the manufacturer's recommendations, the mass range is 2000 to 20000 Da, and the laser intensity remains constant. The EX-Accuspec software and information base were used to analyze the mass spectrum. The quality of protein extraction is assessed by data counts defined by the number of interpretable peaks considered in the algorithm. If the identification score criteria used are more than or equal to 2.0 recommended by the manufacturer, the identification of species level is possible. The higher the score, the higher the confidence of the species level. The score between 1.7 and 2.0 is the possible genus level identification, and the higher the score, the higher the genus level confidence; If the score is less than 1.7, the identification result is not believed, that is, the identification is not reliable and needs to be repeated.

### Primary screening of hvKP

A total of 587 KP strains were collected from the clinical laboratory of A tertiary hospital of traditional Chinese medicine from June 2022 to February 2023. The clinical data of patients from the strains were retrospectively studied to screen the hvKP strains for research. There were 368 males and 219 females, with an average age of 82 years. Referring to previous studies [10, 20], we established the admission criteria for hvKP strain: Isolated KP strains in patients with clinical syndromes that tissue invasive infection (e.g., liver and extrahepatic abscesses, necrotizing fasciitis or endophthalmitis), present multiple sites of infection and/or a tendency to develop subsequent metastatic spread are tentatively defined as hvKP and vice versa as cKP.

### Identification of hvKP

#### Biomarkers for PCR analysis

Based on previous studies, specific biomarker genes of hvKP were analyzed by PCR [8]. For each reaction, 5 ml of 2 × TaqFrogga Mix (Baori Doctor Physical Technology Company, Beijing, China), 0.75 ml of forward primer, 0.75 ml of reverse primer (20 pmol/ml) (Shenggong Biological Engineering Company, Shanghai, China), 1 μl of genomic DNA (50 ng/ml) and 2.5 ml of water were added. PCR was performed under the following cycling conditions: Step 1, 95.0 °C for 2 min; Step 2, 95.0 °C for 30 s; Step 3, primer-specific annealing temperature for 30 s; Step 4, hold for 1 min at 72 ℃; Step 5, repeat steps 2 to 4 for 24 cycles; Step 6, 10 min at 72.0 ℃; Step 7, remain at 4 °C. The PCR amplification products were resolved on a 2% agarose gel (Shenggong Biological Engineering Company, Shanghai, China). The amplification products were observed if they contained the correct DNA sequence. Subsequently, the gene was considered present if a band of the predicted size was detected. Specific primers, PCR, conditions, and product sizes are shown in Table 4.

**Table 4:** Primers, product sizes and PCR conditions of biomarkers [8]

Serial number	Gene	Primer sequence (5, → 3,)	Length (bp)	References
1	_*p*_*rmpA-*F	ACTGGGCTACCTCTGCTTCA	536	[9]
	_*p*_*rmpA*-R	CTTGCATGAGCCATCTTTCA		
2	*iroB*-F	CCCTGGCATATCAAAGGCGT	534	[9]
	*iroB*-R	GACAACAACGCGGGCATTTA		
3	*peg-344*-F	TGGGGTTATTCTTTCGCT	508	[9]
	*peg-344*-R	TTTCCAAGCTTACTGCAATT		
4	_*p*_*rmpA*2-F	GTGCAATAAGGATGTTACATTA	430	[10]
	_*p*_*rmpA*2-R	GGATGCCCTCCTCCTG		
5	*iucA*-F	GCTTATTTCTCCCCAACCC	583	[10]
	*iucA*-R	TCAGCCCTTTAGCGACAAG		

#### SP Determination

##### Qualitative plate SP production test

As previously described [33], Kings B agar plates containing chrome azurol S dye (CAS) were prepared (Xinyu Biotechnology Company, Shanghai, China). Individual colonies of each experimental strain were picked from overnight growing agar plates using 10 μl pipettes, punctured into Kings B agar and incubated at 37 °C. After overnight growth, the formation of an opaque golden yellow area around the colony indicates high-level SP generation.

##### SP quantitative analysis

Referring to a previous study [14], the SP standard deferoxamine mesylate (McLean Biochemical Technology Company, Shanghai, China) containing 0, 1.5, 3.1, 6.25, 12.5, 25, 50 and 100 μg/ml was prepared. The SP analysis solution consisted of 50 ml of 1.2 mM cetyl trimethyl ammonium bromide, 7.5 ml of 2 mM CAS, 1.5 ml of 1 mM FeCl_3_.6H_2_O (Shenggong Biological Engineering Company, Shanghai, China) in 10 mM HCl, and 1.37 M piperazine (McLean Biochemical Technology Company, Shanghai, China) (HCl for pH, adjusted to 5.6). In a flat bottom 96-well plate, 100 μl of each standard or sample was added to the well, then 100 μl of 98% SP was added in duplicate, analyzing the solution and 2% 0.2 M 5-sulfoalicyclic acid solution. The reaction mixture was gently incubated for 30 min, and the results were read at 630 nm. For quantitative analysis, the reference curve is calculated as follows: (OD standard / OD zero standard) × 100. Curves were generated using the Cubic-spline analysis in the Prism software. The SP concentration in each sample was derived from the linear portion of the reference curve. The SP concentration was reported at μg / ml / 1 × 10^9^ CFU.

KP strains that passed our clinical inclusion criteria, which positive in either *peg-344*, *iroB*, *iucA*, *prmpA* or *prmpA2*, and or SP yield greater than 30 μg / ml were defined as hvKP. Conversely, no positive biomarkers, even after passing our clinical inclusion criteria, were defined as cKP.

##### Infection model of Galleria mellonella

The bacterial solution was inoculated into the blood agar plate in four lines and cultured at 37 ℃ for 24 h. A single colony was selected and transferred to an EP tube containing 5 ml M9 medium (Baoxin biological reagent company, Xinjiang, China) and shaken at a constant temperature of 37 ℃ at 200 rpm/min for 8–14 h. The prepared bacterial solution with a concentration of 1 × 10^8^ CFU/mL was diluted with PBS buffer to 1 × 10^7^ CFU/mL, 1 × 10^6^ CFU/mL and 1 × 10^5^ CFU/mL for later use [9]. The experimental animal, Galleria mellonella (Laughing Monkey Information Technology Company, Chongqing, China) weighing 250-350 mg, length of 20-30 mm, with cream color and no gray mark on the body. Store it in a dark environment of 4 °C. Larvae with weak motility or black and gray spots were screened out before the experiment. A 25 μl microsampler (High pigeon Industry and Trade Company, Shanghai, China) was used to absorb 10 μl bacterial solution with different concentrations and inject it into the larvae through the first abdominal segment of the right side of the moth. The injection is performed in a soft, fast and accurate manner to avoid mechanical damage that could affect the experimental results. Each concentration of each strain was injected into 10 larvae. For each batch of experiments, a negative control group which injected with 10 μl PBS buffer and a blank group with no treatment of the insect body should be set up. The injected larvae were placed separately in a blank petri dish, the strain number was marked on the lid of the petri dish, and all the petri dishes were incubated in an incubator at 37℃ for 144 h. After injection, the health status of the larva was recorded at 6 h, 12 h, 24 h, 48 h, 72 h, 96 h, 120 h, 144 h: ① Scores of activity, melanogenesis, cocoon formation and survival of Galleria mellonella at different time points and concentrations (points, x ± s); ② The death time of 80% larvae (LT80) at the same concentration was recorded, and the death of the insect body was manifested as blackening of the body and no response to touch; ③ The survival curve of the larva of the moth was calculated. In the course of the experiment, the dead worms and silk in the plate were cleaned up in time, and the infected moth was treated with a sealed bag and autoclave for harmless treatment.

### Statistical analysis

Microsoft Office 2016 was used to analyze the clinical data of KP, statistical software SPSS 26.0 was used to analyze the research data, the counting data was represented by the number of use cases, the sample rate was compared by the 2 test or Fisher exact probability method, and the measurement data was represented by the mean. T test was used to compare the mean between the two groups, and one-way analysis of variance was used for the three groups and above, *P* < 0.05 indicates a statistically significant difference. Cubic spline analysis in GraphPad 5.0 software was used to generate the standard curve of SP quantitative experiment, and the survival curve and other statistical graphs were drawn.

### Supplementary Information

**Additional file 1: Figure S1.** The Southern blots of five virulence genes. **Table S1.** The SP concentration of hvKP.

## Abbreviations

hvKP: Hypervirulent Klebsiella pneumoniae
CR-hvKP: Carbapenem-resistant hypervirulent Klebsiella pneumoniae
CS-hvKP: Carbapenem-sensitive hypervirulent Klebsiella pneumoniae
KP: Klebsiella pneumoniae
cKP: Classical Klebsiella pneumoniae
SP: Siderophores
PCR: Polymerase chain reaction
MLST: Multilocus sequence typing
PFGE: Pulse field gel electrophoresis
PBS: Phosphate buffer saline
