# Supplementary material for: Identification of hypervirulent Klebsiella pneumoniae based on biomarkers and Galleria mellonella infection model
Source: BMC Microbiol. 2023 Nov 29;23:369. doi: 10.1186/s12866-023-03124-0 (PMC10685466; doi:10.1186/s12866-023-03124-0)
Supplement: Supplementary file 1 — Additional file 1: Figure S1. The Southern blots of five virulence genes. Table S1. The SP concentration of hvKP. [file 12866_2023_3124_MOESM1_ESM.docx]

**Supplementary Information**

Figure S1 The Southern blots of five virulence genes.


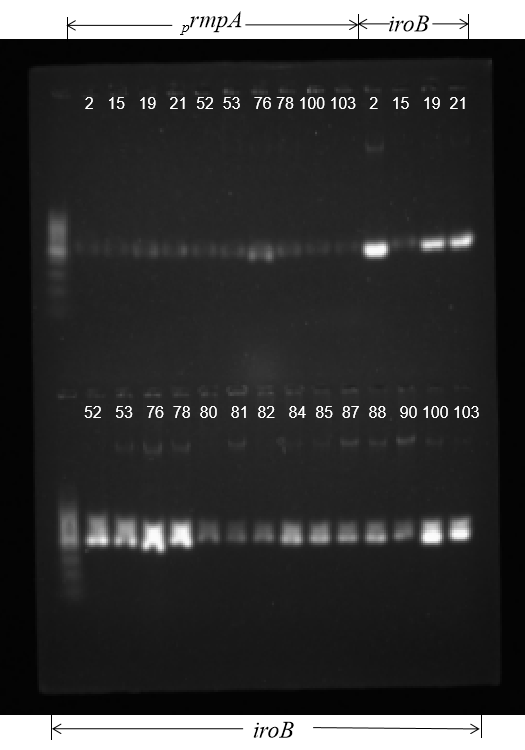


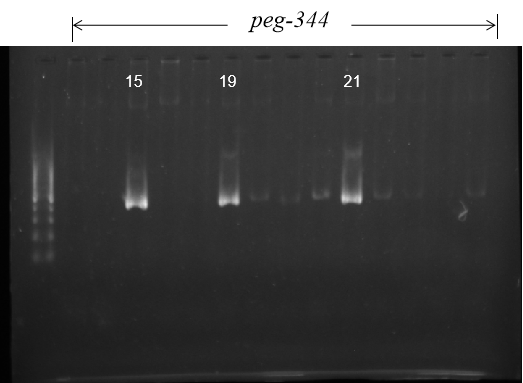


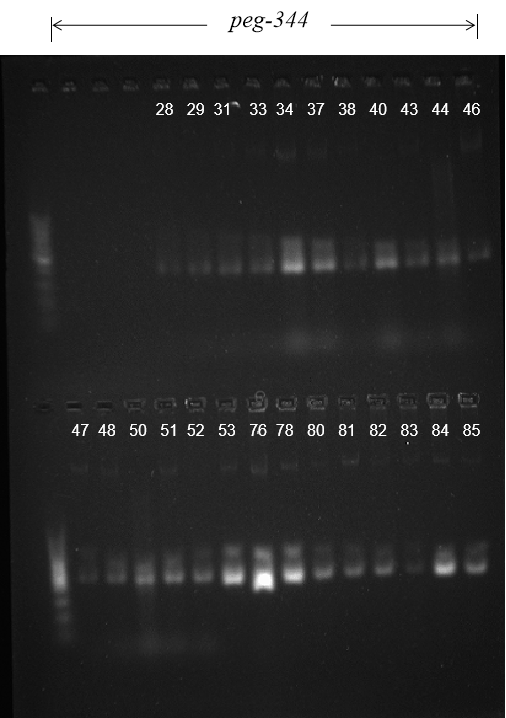


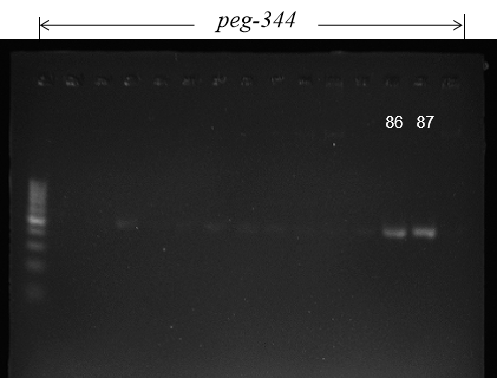


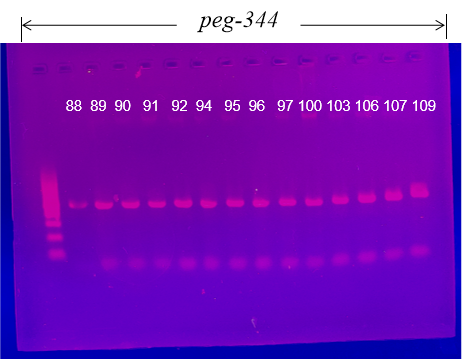


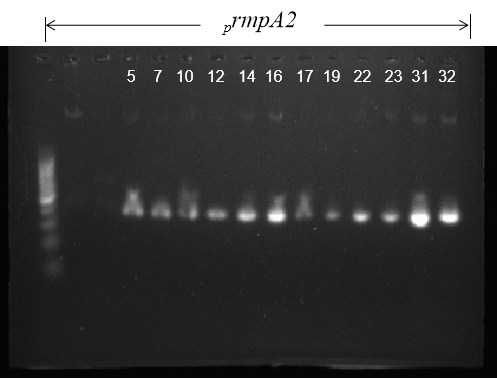


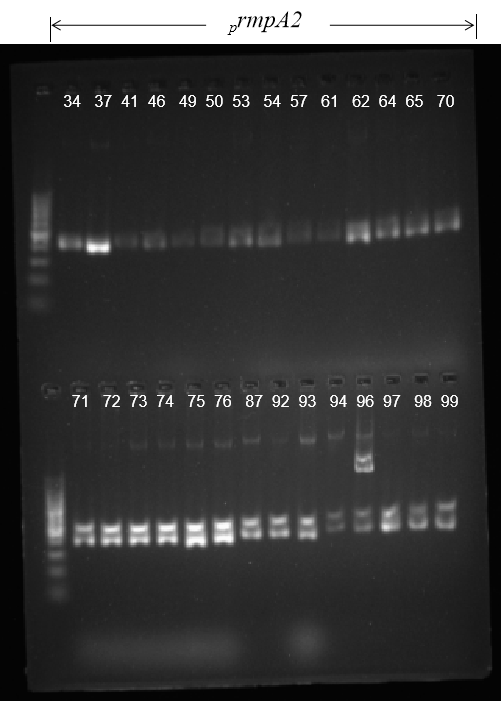


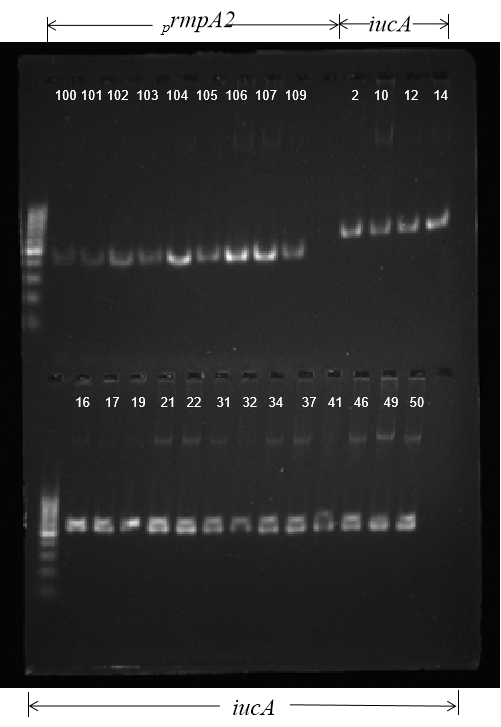


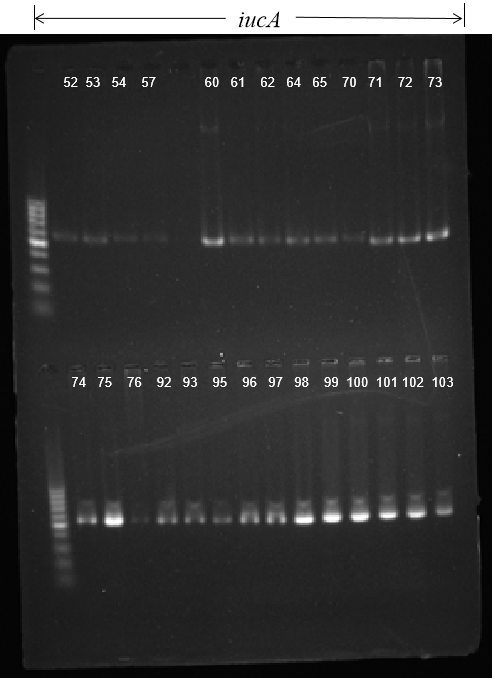


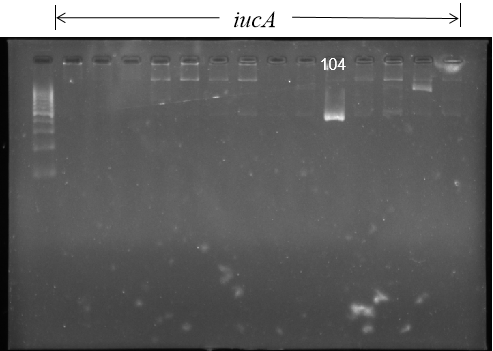


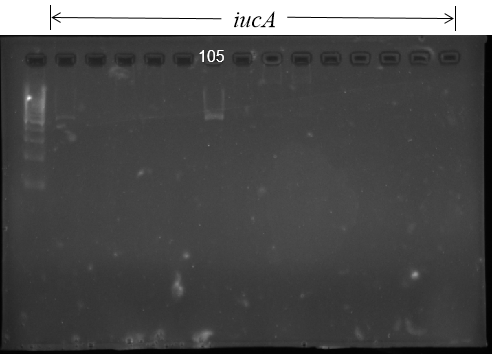


Table S1 The SP concentration of hvKP

| Serial number | Strain number | OD | SP浓度(μg/ml) | OD600/109CFU/ml | 109CFU | μg/ml/109CFU |
| --- | --- | --- | --- | --- | --- | --- |
| 1 | 7 | 0.118 | 8.559 | 1.23 | 0.123 | 69.585 |
| 2 | 16 | 0.115 | 9.009 | 1.148 | 0.115 | 78.476 |
| 3 | 20 | 0.136 | 6.607 | 1.226 | 0.123 | 53.891 |
| 4 | 22 | 0.108 | 10.36 | 1.094 | 0.109 | 94.698 |
| 5 | 25 | 0.127 | 7.508 | 1.233 | 0.123 | 60.892 |
| 6 | 26 | 0.116 | 8.859 | 1.232 | 0.123 | 71.907 |
| 7 | 28 | 0.127 | 7.508 | 1.261 | 0.126 | 59.540 |
| 8 | 29 | 0.098 | 13.163 | 1.168 | 0.117 | 112.697 |
| 9 | 30 | 0.143 | 6.006 | 1.18 | 0.118 | 50.898 |
| 10 | 31 | 0.156 | 5.105 | 1.255 | 0.126 | 40.677 |
| 11 | 33 | 0.138 | 6.406 | 1.205 | 0.121 | 53.162 |
| 12 | 35 | 0.124 | 7.808 | 1.132 | 0.113 | 68.975 |
| 13 | 36 | 0.144 | 5.906 | 1.178 | 0.118 | 50.136 |
| 14 | 37 | 0.106 | 10.761 | 1.181 | 0.118 | 91.118 |
| 15 | 39 | 0.138 | 6.406 | 1.097 | 0.110 | 58.396 |
| 16 | 40 | 0.118 | 8.559 | 0.408 | 0.041 | 209.779 |
| 17 | 41 | 0.112 | 9.56 | 1.219 | 0.122 | 78.425 |
| 18 | 42 | 0.124 | 7.808 | 1.119 | 0.112 | 69.777 |
| 19 | 43 | 0.135 | 6.707 | 1.231 | 0.123 | 54.484 |
| 20 | 44 | 0.123 | 7.908 | 1.293 | 0.129 | 61.160 |
| 21 | 46 | 0.11 | 9.86 | 1.225 | 0.123 | 80.490 |
| 22 | 48 | 0.132 | 7.007 | 1.861 | 0.186 | 37.652 |
| 23 | 49 | 0.131 | 7.107 | 1.245 | 0.125 | 57.084 |
| 24 | 50 | 0.136 | 6.607 | 1.757 | 0.176 | 37.604 |
| 25 | 54 | 0.127 | 7.508 | 1.214 | 0.121 | 61.845 |
| 26 | 55 | 0.136 | 6.607 | 1.28 | 0.128 | 51.617 |
| 27 | 56 | 0.135 | 6.707 | 1.201 | 0.120 | 55.845 |
| 28 | 58 | 0.140 | 6.306 | 1.157 | 0.116 | 54.503 |
| 29 | 59 | 0.131 | 7.107 | 1.105 | 0.111 | 64.317 |
| 30 | 60 | 0.114 | 9.159 | 1.233 | 0.123 | 74.282 |
| 31 | 61 | 0.112 | 9.56 | 1.128 | 0.113 | 84.752 |
| 32 | 62 | 0.129 | 7.307 | 1.18 | 0.118 | 61.924 |
| 33 | 63 | 0.137 | 6.507 | 1.153 | 0.115 | 56.435 |
| 34 | 64 | 0.12 | 8.308 | 1.293 | 0.129 | 64.254 |
| 35 | 65 | 0.086 | 19.119 | 2.036 | 0.204 | 93.905 |
| 36 | 67 | 0.132 | 7.007 | 1.249 | 0.125 | 56.101 |
| 37 | 68 | 0.138 | 6.406 | 1.148 | 0.115 | 55.801 |
| 38 | 70 | 0.12 | 8.308 | 1.17 | 0.117 | 71.009 |
| 39 | 71 | 0.111 | 9.71 | 1.199 | 0.120 | 80.984 |
| 40 | 72 | 0.115 | 9.009 | 1.21 | 0.121 | 74.455 |
| 41 | 73 | 0.112 | 9.56 | 1.163 | 0.116 | 82.201 |
| 42 | 74 | 0.095 | 14.364 | 1.133 | 0.113 | 126.778 |
| 43 | 75 | 0.116 | 8.859 | 1.199 | 0.120 | 73.887 |
| 44 | 76 | 0.097 | 13.564 | 1.101 | 0.110 | 123.197 |
| 45 | 77 | 0.106 | 10.761 | 1.238 | 0.124 | 86.922 |
| 46 | 79 | 0.153 | 5.305 | 1.229 | 0.123 | 43.165 |
| 47 | 82 | 0.126 | 7.608 | 1.09 | 0.109 | 69.798 |
| 48 | 87 | 0.101 | 12.212 | 1.2 | 0.120 | 101.767 |
| 49 | 89 | 0.133 | 6.907 | 1.4 | 0.140 | 49.336 |
| 50 | 91 | 0.133 | 6.907 | 1.217 | 0.122 | 56.754 |
| 51 | 95 | 0.102 | 11.912 | 1.197 | 0.120 | 99.515 |
| 52 | 96 | 0.112 | 9.56 | 1.131 | 0.113 | 84.527 |
| 53 | 98 | 0.104 | 11.311 | 1.231 | 0.123 | 91.885 |
| 54 | 99 | 0.134 | 6.807 | 1.293 | 0.129 | 52.645 |
| 55 | 101 | 0.089 | 17.167 | 1.157 | 0.116 | 148.375 |
| 56 | 103 | 0.123 | 7.908 | 1.201 | 0.120 | 65.845 |
| 57 | 105 | 0.09 | 16.617 | 1.242 | 0.124 | 133.792 |
| 58 | 107 | 0.113 | 9.359 | 1.212 | 0.121 | 77.219 |
| 59 | 109 | 0.089 | 17.167 | 1.138 | 0.114 | 150.852 |
